# Efficacy of Two Monoterpenoids, Carvacrol and Thymol, and Their Combinations against Eggs and Larvae of the West Nile Vector *Culex pipiens*

**DOI:** 10.3390/molecules24101867

**Published:** 2019-05-15

**Authors:** Mohammad Reza Youssefi, Mohaddeseh Abouhosseini Tabari, Aryan Esfandiari, Sohrab Kazemi, Ali Akbar Moghadamnia, Stefania Sut, Stefano Dall’Acqua, Giovanni Benelli, Filippo Maggi

**Affiliations:** 1 Department of Veterinary Parasitology, Babol-Branch, Islamic Azad University, Babol 484, Iran; youssefi929@hotmail.com; 2 Faculty of Veterinary Medicine, Amol University of Special Modern Technologies, Amol 46131-46391, Iran; m_abuhoseini@yahoo.com; 3 Young Researchers and Elite Club, Babol Branch, Islamic Azad University, Babol 484, Iran; aryan22.es@gmail.com; 4 Cellular and Molecular Biology Research Center, Health Research Center, Babol University of Medical Sciences, Babol 47176-47745, Iran; kazemi.msm@gmail.com; 5 Department of Agronomy, Food, Natural Resources, Animals and Environment (DAFNAE), University of Padova, 35020 Legnaro, Italy; stefania_sut@hotmail.it; 6 Department of Pharmaceutical and Pharmacological Sciences, University of Padova, 35139 Padova, Italy; 7 Department of Agriculture, Food and Environment, University of Pisa, via del Borghetto 80, 56124 Pisa, Italy; 8 School of Pharmacy, University of Camerino, 62032 Camerino, Italy; filippo.maggi@unicam.it

**Keywords:** biopesticide, essential oil, ovicidal activity, larvicidal activity, mosquito vector

## Abstract

*Background*: Insect vector control is facing the challenges of resistance development and environmental hazards caused by synthetic pesticides. This has led to a considerable market opportunity for botanical insecticides. In this scenario, our study investigated the potential of selected bioactive monoterpenoids, carvacrol and thymol, as safe and effective tools to control the West Nile vector *Culex pipiens*. Furthermore, the combined effect of thymol-carvacrol mixtures and their possible interactions were assessed. *Methods*: For determining larvicidal and ovicidal 50% lethal concentration (LC_50_), each monoterpenoid was tested at different concentrations (5–500 mg/L). Then, the fixed ratio method was used for evaluating their combinational efficacy. *Results*: Carvacrol was more toxic against larvae of *Cx. pipiens,* with a LC_50_ value of 14 mg/L, whereas thymol exhibited a LC_50_ value of 49 mg/L. Comparable trends of efficacy were observed when toxicity on *Cx. pipiens* eggs was investigated, with LC_50_ values of 7 and 13 mg/L for carvacrol and thymol, respectively. In combinational toxicity assays, the mixture thymol-carvacrol at 1:4 ratio achieved a synergistic effect against larvae of *Cx. pipiens,* whereas an additive effect was observed on eggs. Other ratios showed antagonistic effects. *Conclusions*: Overall, our findings pointed out that the 1:4 ratio of thymol-carvacrol blend can enhance the insecticidal efficacy on *Cx. pipiens* young instars and can be considered further as active ingredient for developing botanical insecticides to be used in mosquito control operations.

## 1. Introduction

The effective and environmentally sustainable management of arthropod vectors is of great relevance and has attracted the attention of researchers for centuries [1,2,3,4]. Mosquitoes (Diptera: Culicidae) are currently recognized as the most important vectors in terms of public health importance, playing a crucial role in the spread of malaria, yellow fever, dengue, West Nile, Rift Valley fever, Japanese encephalitis, chikungunya and Zika virus, just to cite a few examples [5,6]. 

However, concerning mosquito control programs, several challenges still need to be faced [6,7], including the quick development of insecticide resistance in targeted vector populations [8,9], as well as severe non-target effects of synthetic pesticides on human health and the environment [10]. A possible route to tackle this challenge is the development of novel pesticides based on plant secondary metabolites, which are characterized by multiple modes of action [11,12,13,14]. Indeed, in recent years, researchers have attempted to find out new sources of safe and eco-friendly plant-based insecticides and acaricides [15,16,17]. In this framework, a significant number of studies provides interesting insights into the efficacy of plant extracts and essential oils as ovicides and larvicides against many mosquito vectors of medical and veterinary importance [18,19]. 

However, plant essential oil composition can vary consistently according to many factors, including botanical species, geographical area of origin, growing conditions, genetic variability, harvesting time and extraction technique, just to cite some of the main ones [20]. This can play a major impact on their insecticidal activity, making the development of products for real-world use challenging. Therefore, an approach to deal with this issue is the detection of the bioactive components from plant essential oils, and their encapsulation or micro- and nanoformulations for highly stable pesticide development [21,22,23,24,25].

Ten-carbon components of plant essential oils as monoterpenoids have been widely recognized as toxic, repellent, and antifeedant agents on insect pests and vectors. Thus, they are considered as potential molecules for the development of novel and eco-friendly pesticides [26,27]. Monoterpenoids are synthesized in the cytoplasm and plastids of the plant cell through two distinct pathways, namely mevalonate and methyl erythritol phosphate, respectively [28]. They are endowed with a multitude of different chemical structures (e.g., linear, monocyclic, bicyclic) and functional groups (e.g., double bonds, alcoholic, aldehydic, ketonic, phenolic). Among them, phenolic monoterpenes as thymol and carvacrol have been recognized as two of the most powerful bioactive constituents produced by higher plants [29]. Research on the toxicity of these phenolic monoterpenes on various insect pests have highlighted their potential as ovicides, fumigants and contact toxicants [30]. Carvacrol is a monoterpene phenol that occurs in many essential oils of the Lamiaceae family, including *Origanum*, *Satureja*, *Thymbra*, *Thymus*, and *Coridothymus* species [31,32]. Its isomer thymol can be found in high amounts in essential oils of *Thymus* species, *Ocimum gratissimum* L. and *Trachyspermum ammi* (L.) Sprague [33,34,35,36,37]. Carvacrol and thymol were reported to have broad insecticidal activity against arthropod species of agricultural, medical and veterinary importance [23,38,39,40,41], including *Anopheles, Aedes* and *Culex* mosquitoes [42,43,44,45]. Noteworthy, these compounds are recognized as Generally Recognized as Safe (GRAS) by the FDA and EPA and have LD_50_, values as determined in rats after oral administration, of around 1 g/kg, making their formulations likely devoid of toxic effects on humans and animals.

To our knowledge, no data are available on the efficacy of these two monoterpenes and their mixtures against the mosquito species *Culex pipiens* L. The latter is part of the *Culex pipiens* complex, whose species, found worldwide in urban and sub-urban areas of temperate and tropical regions, vector important pathogens including West Nile and St. Louis encephalitis virus, among others, as well as parasites like lymphatic filariasis and avian malaria [46]. In particular, since West Nile is expanding its geographical range in Europe causing an increasing number of epidemics/outbreaks [47], the effective management of *Cx. pipiens* mosquitoes under the Integrated Vector Management (IVM) is timely and important. Thus, to develop novel insecticides of botanical origin characterized by a multiple mechanism of action [11,48] and active at very low doses [14], the present study was conducted to assess the toxicity of carvacrol and thymol on eggs and larvae of *Cx. pipiens*. Furthermore, when formulating selected plant essential oil constituents in insecticidal blends, an important issue needing attention is the possible synergistic and antagonistic effects of the blend, as outlined by research conducted on various arthropod species of economic importance, including agricultural pests [49], houseflies [50], mites [51], mosquitoes [22] and ticks [41]. Therefore, in our study, the combined effect of thymol-carvacrol and their possible interaction were assessed on both eggs and larvae of *Cx. pipiens* mosquitoes, using the fixed ratio method.

## 2. Results

**Figure 1:**
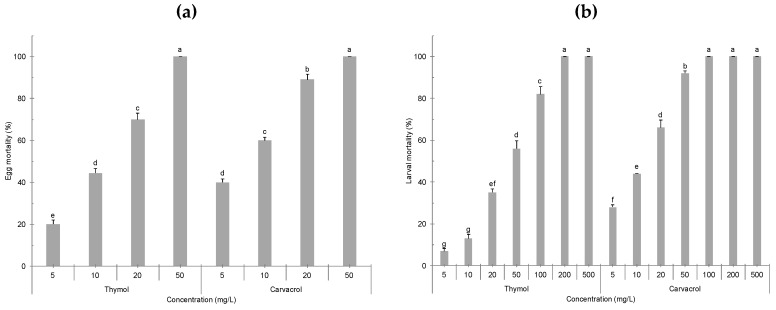
Insecticidal activity of the selected monoterpenoids carvacrol and thymol at different concentrations against eggs (**a**) and 3rd instar larvae (**b**) of *Culex pipiens* mosquitoes after 24 h. T-bars indicate standard errors; above each column, different letters indicate significant differences among means (ANOVA, Tukey’s HSD test, *p* < 0.05).

*Culex pipiens* egg mortality was recorded after 24 h exposure to different concentrations of carvacrol and thymol (Figure 1a). A significant effect of the tested compound (*F_1,32_* = 96.121, *p* < 0.0001), the concentration (*F_3,32_* = 708.862, *p* < 0.0001), and their interaction (*F_3,32_* = 11.949, *p* < 0.0001) was observed. At the minimum tested concentration of 5 mg/L, both thymol and carvacrol resulted in 20.2 and 40.4% decrease in egg hatchability, respectively, while 50 mg/L led to 100% egg mortality (Figure 1a). Overall, carvacrol achieved LC_50_ and LC_90_ values of 7 and 20 mg/L, respectively, showing higher toxicity on mosquito eggs if compared with thymol, which had LC_50_ and LC_90_ values of 13 and 27 mg/L, respectively (Table 1).

Larvicidal assays also showed a significant effect of the tested compounds (*F_1,56_* = 472.971, *p* < 0.0001), the concentration (*F_6,56_* = 885.684, *p* < 0.0001), and their interaction (*F_6,56_* = 34.418, *p* < 0.0001). As shown in Figure 1b, after 24 h of exposure to 50 mg/L of carvacrol, 92% mosquito mortality was achieved, while thymol tested at the same concentration led to 52% mortality of *Cx. pipiens* larvae. Concerning probit analysis conducted on larvicidal results, carvacrol showed LC_50_ and LC_90_ values of 14 and 44 mg/L, respectively, while thymol achieved LC_50_ and LC_90_ values of 49 and 112 mg/L, respectively (Table 1).

**Figure 2:**
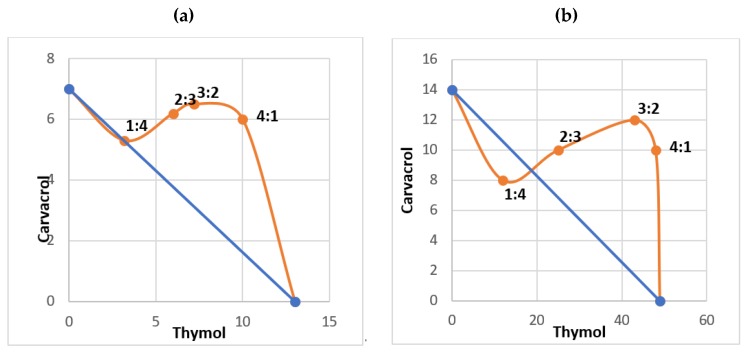
Isobologram of the interaction between carvacrol and thymol in binary combinations using the fixed ratio method: activity on eggs (**a**) and 3rd instar larvae (**b**) of *Culex pipiens*.

Results of the quantitative analysis of LC_50_ combinational toxicity of carvacrol and thymol and their FLC indices are provided in Table 2. The trend of interaction between thymol-carvacrol combinations against eggs and 3rd instar larvae of *Cx. pipiens* was analysed by the fixed ratio method and the obtained isobolograms were given in Figure 2. The 1:4 ratio of thymol-carvacrol caused the highest toxicity, showing a synergistic effect against larvae of *Cx. pipiens* with FLC index of 0.79, whereas an additive effect was observed (FLC = 1) against eggs (Figure 2). Other ratios led to antagonistic effects.

## 3. Discussion

### 3.1. Ovicidal and Larvicidal Efficacy

Monoterpenoids are among the most important bioactive compounds in essential oil research. Concerning the development of novel insecticides, they have been reported as effective against a wide variety of species of medical, veterinary and agricultural importance, highlight their efficacy as ovicides, larvicides and even adulticides [21,22,23,26,27]. The present study highlighted the promising potential of carvacrol and thymol as ovicidal and larvicidal agents against the West Nile vector *Cx. pipiens*. To the best of our knowledge, little has been reported on these two compounds as mosquito ovicides. On the other hand, our larvicidal results substantiated the earlier report by Traboulsi et al. [42], who highlighted the toxicity of eight components of plant essential oils against 4th instar larvae of *Cx. pipiens molestus*. Among the eight tested compounds, carvacrol and thymol were the most toxic ones. However, against this latter mosquito, they achieved LC_50_ values of 36 and 37.6 mg/L, respectively [42]; carvacrol LC_50_ value was substantially higher if compared to probit results achieved by the same compound in our study on *Cx. pipiens* 3rd instar larvae (LC_50_ of 14 mg/L).

Besides, the larvicidal activity of carvacrol, the major constituent of *O. vulgare* L. essential oil, has been evaluated against 3rd stage larvae of other mosquito species. Carvacrol has been reported effective against *Anopheles stephensi* Liston, with a LC_50_ value of 21.15 μg/mL, and exhibited LC_50_ values of 26.08 and 27.95 μg/mL on *Cx. quinquefasciatus* Say and *Cx. tritaeniorhynchus* Giles, respectively [44]. Knio et al. [52] reported LC_50_ of 35.5 and 33.7 ppm for carvacrol and thymol against larvae of the seaside mosquito, *Aedes* (*Ochlerotatus*) *caspius* (Pallas). Finally, Carvalho et al. [53] evaluated the larvicidal activity of the essential oil from *Lippia sidoides* Cham., and its two major components, carvacrol and thymol, against *Aedes aegypti* L. Thymol caused 100% mortality even at 0.017 % concentration. However, in contrast to our findings on *Cx. pipiens* and to the studies detailed above, these authors failed to observe any mosquito larvicidal activity for carvacrol tested at 0.04 % [53].

### 3.2. Ovicidal and Larvicidal Efficacy of the Blend Containing Carvacrol and Thymol

The increasing levels of insecticide resistance in targeted arthropod populations worldwide [8,9] require highly effective strategies to establish reliable vector control methods within the IVM framework, with proven epidemiological impact [1]. The employ of botanical-based insecticidal blends as well as combinations of synthetic or bacterial-borne (e.g., toxins from *Bacillus thuringiensis* Berliner serovar. *israelensis*) pesticides with plant secondary metabolites can be a promising strategy to reduce the development of resistance [19,54], relying on components with multiple mechanisms of action [54,55].

Overall, decreasing the risk of pesticide resistance development, reducing the employed concentrations, and exploiting the synergistic actions between selected molecules are some of the main advantages of combining phytochemicals [31,37,56,57]. Shaalan et al. [55] showed that binary mixtures of selected botanical extracts were more effective than non-mixed ones against 4th instar larvae of *Ae. aegypti* and *Cx. annulirostris* Skuse. Besides mosquitoes, Hummelbrunner and Isman [58] earlier evaluated the toxicity of some monoterpenoids commonly found in plant essential oils on the moth pest *Spodoptera litura* Fabr. (Lepidoptera: Noctuidae). They showed synergistic acute toxicity and feeding deterrence of (*E*)-anethole with thymol, citronellal, and (*R*)-terpineol, and suggested synergistic 1:1 mixtures for development of effective control agents since the use of smaller amounts in the mixture resulted in achieving satisfactory levels of efficacy [58]. In agreement with these authors, our results revealed enhanced activity of the thymol-carvacrol mixture on larvae of *Cx. pipiens,* when compared to the bioactivity of individual components tested alone.

The fixed ratio method and isobologram construction showed different interactions of the two monoterpene phenols when administered in combination. The most efficient ratio of thymol-carvacrol was 1:4, which resulted in synergistic effect against larvae and additive effect against eggs of *Cx. pipiens*. The various physiological interactions of plant-borne products with different insect targets is a complex issue to deal with. Concerning our findings, we may hypothesize that the 1:4 thymol-carvacrol combination is able to completely inhibit some receptors (e.g., octopamine and GABA ones) causing to insect neurotoxicity, or is the most effective in interacting with more than one target, leading to synergistic effects. Further research on this is warranted, since studies on the molecular effects of synergistic mixtures are extremely limited. More generally, when searching literature about the synergistic effects of natural products in mosquito ovicidal studies we faced a severe lack of literature. On the other hand, important research has been done on the essential oil toxicity to mosquito larvae [31,37,55] and pure compounds [19]. Concerning the latter category, Pavela tested binary mixtures of 30 selected essential oil constituents on *Cx. quinquefasciatus* larvae showing that thymol was the most effective, with a LC_50_ of 18 mg/L, while carvacrol was among the 9 substances showing synergy with >20 compounds [18]. It is noteworthy that carvacrol and thymol tested in a binary blend had a synergistic effect on *Cx*. *quinquefasciatus*, as also highlighted by our results on *Cx*. *pipiens*. However, in the study by Pavela [19], the highest larvicidal synergy on *Cx*. *quinquefasciatus* was observed testing carvacrol + carvone, carvacrol + 4-allylanisole, and carvacrol + terpineol, among others. Besides, the author outlined, as shown for carvacrol + thymol on *Cx. pipiens* data presented here, that some of the most effective mixtures varied in their efficacy according to the mixing ratio. In particular, 100% larvicidal efficacy was obtained formulating carvacrol + carvone at a ratio >2. Overall, Pavela et al. [24] selected two binary mixtures leading to mortality > 90% when tested at <20 mg/L: limonene + (*E*)-anethole (1:1) and carvacrol + carvone (1:2–3). In contrast to our findings, Karpouhtsis et al. [59] reported that mixing carvacrol and thymol, resulted in reduction of insecticidal activity on *Drosophila melanogaster* Meigen (Diptera: Drosophilidae), suggesting an antagonistic interaction between them. It was observed that the toxicity of carvacrol was reduced in presence of thymol. Interestingly, the obtained data and the trend of isobologram curve in the present study pointed out a loss of synergy when increasing the concentration of thymol and decreasing that of carvacrol. In other words, adding thymol to carvacrol, especially in the combinations containing higher amounts of thymol over carvacrol, can result in antagonistic interactions between these two molecules (FLC > 1).

Synergistic activity of carvacrol combined with thymol has also been reported recently for mite and tick species. Indeed, carvacrol + thymol particularly in a 4:1 ratio led to high acaricidal activity against the poultry red mite, *Dermanyssus gallinae* (De Geer) [27]. Furthermore, according to Novato et al. [41] thymol + carvacrol at a 1:1 ratio of LC_50_ achieved synergistic effect towards larvae of *Dermacentor nitens* Neumann and *Amblyomma sculptum* Berlese, while at 1/4 LC_50_ showed an additive effect against *D. nitens* [41]. Araujo et al. [60] also evaluated, among others, the synergistic effect of thymol and carvacrol in larvae of the cattle tick, *Rhipicephalus (Boophilus) microplus* (Canestrini), and brown dog tick, *Rhipicephalus sanguineus* (Latreille). They reported that combinations of carvacrol + thymol, carvacrol + eugenol and thymol + eugenol had synergistic effects against *R. microplus* and *R. sanguineus s.l*. larvae [60].

The toxicity of carvacrol and thymol to arthropod pests and vectors can be due to different mechanisms. Research has suggested that carvacrol and thymol potentiate ligand-gated chloride channels in insect nervous system and probably act as neurotoxic insecticides [48,61]. Furthermore, it was shown that thymol may work by blocking octopamine receptors, which are unique to insects and considered as an important target site for pest and vector control [10,14]. On the other hand, the actual role of thymol and carvacrol to interact with the cholinergic system of insects cannot be neglected [62]. Instead, their interaction with the detoxification system of insects can be excluded [63].

Based on these findings, only the thymol-carvacrol ratio 1:4 in the mixture was found effective in producing synergistic toxicity in young instars. The latter may be given by the concomitant multiple mode of actions of the two compounds that surely deserves further investigation.

## 4. Materials and Methods

### 4.1. Chemicals

Carvacrol and thymol were purchased from Sigma-Aldrich (Steinheim, Germany) and stored in a sealed brown container until bioassays. All other chemicals were analytical grade and commercially available.

### 4.2. Ovicidal and Larvicidal Assays

A laboratory strain of *Cx. pipiens* was reared as described by Tabari et al. [22]. Ovicidal activity was evaluated according to the method of Tabari et al. [22]. Freshly laid egg rafts of *Cx. pipiens* (about 100 eggs per replicate) were submerged for 24 h with water plus different concentrations of carvacrol and thymol (i.e., 5, 10, 20, and 50 mg/L in 5 mL tap water containing 0.4% DMSO). Negative control was water + 0.4% DMSO. 5 replicates were done for each tested concentration and control as well. 24 h post-treatment, egg mortality (%) was estimated as follows:

(1) $$\mathrm{Egg}\;\mathrm{mortality}\;(\%)\;=\;\frac{\mathrm{Total}\;\mathrm{no}.\;\mathrm{of}\;\mathrm{eggs}\;-\;\mathrm{no}.\;\mathrm{of}\;\mathrm{hatched}\;\mathrm{larvae}}{\mathrm{Total}\;\mathrm{no}.\;\mathrm{of}\;\mathrm{eggs}}\;\times \;100$$

Larvicidal activity of carvacrol and thymol was evaluated on 3rd instar larvae according to the WHO protocol [64] with slight modifications [22]. Both carvacrol and thymol were tested at 5, 10, 20, 50, 100, 200, and 500 mg/L in tap water. To formulate the concentrations detailed above, both compounds were dissolved in 1 mL of dimethyl sulfoxide DMSO then diluted in water [24]. Negative control was 1 mL DMSO + 249 mL of water. In each replicate, 20 larvae were tested, 5 replicates were done per each concentration. Larval mortality was noted after 24 h [1].

### 4.3. Combinational Bioassays

This second series of experiments was aimed to determine possible synergistic or antagonistic activity of combination of carvacrol and thymol. Based on the fixed ratio method, four different ratios of thymol-carvacrol, i.e., 4:1, 3:2, 2:3, and 1:4 *v*/*v*, were tested for their combinational toxicity on eggs and larvae of *Cx. pipiens*. The method by Pastor et al. [57] was followed; for each ratio, two-fold serial dilutions were performed to give final concentrations ranging from 1 to 100 mg/L. Three replicates were performed for each combination ratio.

### 4.4. Statistical Analysis

Egg and larval mortality data transformed by arcsine√ were analyzed by ANOVA and Tukey’s HSD test (*p* ≤ 0.05). To determine LC_50_ and LC_90_ values, mortality data, corrected with Abbott’s [65] formula where needed, were subjected to probit analysis [66]. When 95% confidence limits (95% CL) failed to overlap, LC_50_ and LC_90_ values were considered significantly different from one another. Concerning the combination assays, the obtained data were plotted for the construction of an isobologram for both larvicidal and ovicidal activity of thymol-carvacrol combinations. Then, 50% lethal concentration (LC_50_) and the fractional lethal concentration (FLC) index for each ratio were calculated. FLC index was calculated according to the following formula:

FLC index = LC_50_ A in combination/LC_50_ A alone + LC_50_ B in combination/LC_50_ B alone.

An FLC index equal to 1.0 indicates additivity, FLC < 1.0 indicates tendency to synergy, and FLC > 1.0 indicates a trend toward antagonism [67].

## 5. Conclusions and Outlooks for Future Research

Overall, the present investigation firstly shed light on the high toxicity of carvacrol and thymol against mosquito eggs. In addition, outstanding larvicidal activity was observed on *Cx*. *pipiens* larvae, with LC_50_ values lower < 50 mg/L for both molecules. Extremely low LC_50_ were detected both on eggs and larvae of *Cx*. *pipiens*, with values always lower than 20 mg/L. Combinational assays pointed out a synergistic effect testing 1:4 ratio of thymol plus carvacrol. Detecting a synergistic effect in bioactive phytochemical mixtures could be helpful in future operations aimed to manage West Nile and filariasis mosquito vectors exploiting low concentrations of the active ingredients, and their multiple mode of action, which can be helpful to reduce the risk of insecticide resistance development. Lastly, further ecotoxicology research to assess the impact of the binary mixtures proposed here on non-target species, with special reference to chronic toxicity on aquatic organisms, are still needed.

## Figures and Tables

**Table 1:** Probit analysis showing the toxicity of carvacrol and thymol against eggs and 3rd instar larvae of the West Nile vector *Culex pipiens*.

Tested Compound	Targeted Instar	LC_50_ (mg/L)	95% LCL-UCL	LC_90_ (mg/L)	95% LCL-UCL	*χ^2^(df)*
Carvacrol	Egg	7	6-8	20	19-22	2.08 (5) *n.s.*
	3rd instar larva	14	11-17	44	38-52	4.23 (5) *n.s.*
Thymol	Egg	13	12-14	27	25-31	1.56 (5) *n.s.*
	3rd instar larva	49	42-52	112	99-130	2.98 (5) *n.s.*

*n.s.* = not significant (*p* > 0.05).

**Table 2:** Ovicidal and larvicidal results achieved on *Culex pipiens* testing carvacrol-thymol combinations with the fixed ratio method.

Thymol:Carvacrol Ratio	LC_50_ Thymol:Carvacrol in Combination (mg/L) on Eggs	FLC index ^a^ on Eggs	LC_50_ Thymol:Carvacrol in Combination (mg/L) on 3rd Instar Larvae	FLC Index ^a^ on 3rd Instar Larvae
4:1	10:6	1.56	48:10	1.65
3:2	7.2:6.5	1.41	43:12	2.10
2:3	6:6.2	1.28	25:10	1.19
1:4	3.5:5.5	1.00	12:8	0.79

^a^ FLC index = LC_50_ carvacrol in combination/LC_50_ carvacrol alone + LC_50_ thymol in combination/LC_50_ thymol alone.
